# The development of nuclear medicine in Slovenia and Ljubljana; half a century of nuclear medicine in Slovenia

**DOI:** 10.2478/v10019-012-0011-5

**Published:** 2012-01-12

**Authors:** Zvonka Zupanic Slavec, Simona Gaberscek, Ksenija Slavec

**Affiliations:** 1 Institute for the History of Medicine, Faculty of Medicine, University of Ljubljana, Slovenia; 2 Department of Nuclear Medicine, University Medical Centre Ljubljana, Slovenia; 3 Faculty of Medicine, University of Ljubljana, Slovenia

**Keywords:** nuclear medicine, history, Slovenia, Ljubljana, Clinic for Nuclear Medicine

## Abstract

**Background:**

Nuclear medicine began to be developed in the USA after 1938 when radionuclides were introduced into medicine and in Europe after radionuclides began to be produced at the Harwell reactor (England, 1947). Slovenia began its first investigations in the 1950s. This article describes the development of nuclear medicine in Slovenia and Ljubljana. The first nuclear medicine interventions were performed in Slovenia at the Internal Clinic in Ljubljana in the period 1954–1959. In 1954, Dr Jože Satler started using radioactive iodine for thyroid investigations. In the same year, Dr Bojan Varl, who is considered the pioneer of nuclear medicine in Slovenia, began systematically introducing nuclear medicine. The first radioisotope laboratories were established in January 1960 at the Institute of Oncology and at the Internal Clinic. Under the direction of Dr. Varl, the laboratory at the Internal Clinic developed gradually and in 1973 became the Clinic for Nuclear Medicine with departments for *in vivo* and *in vitro* diagnostics and for the treatment of inpatients and outpatients at the thyroid department. The Clinic for Nuclear Medicine became a teaching unit of the Medical Faculty and developed its own post-graduate programme – the first student enrolled in 1972. In the 1960s, radioisotope laboratories opened in the general hospitals of Slovenj Gradec and Celje, and in the 1970s also in Maribor, Izola and Šempeter pri Novi Gorici.

**Conclusions:**

Nowadays, nuclear medicine units are modernly equipped and the staff is trained in morphological, functional and laboratory diagnostics in clinical medicine. They also work on the treatment of cancer, increased thyroid function and other diseases.

## Introduction

Half a century ago, nuclear medicine as a science was not yet known. In this profession, open and artificial radionuclides are used, created in special biochemical substances–radiopharmaceuticals. Radiopharmaceuticals enter the body and emit beams to the radioactivity-meter outside the body, enabling the detection of morphological and functional changes within organs. They are also used for irradiation of diseased tissue and for the measurement of serum concentration of biological substances. In the USA, radionuclides were introduced into medicine after 1938 and in Europe after 1947, when production began at the Harwell reactor in England. Slovenia followed global trends and in the 1950s the first investigations in this field also began in Slovenia. Nuclear medicine units obtained radionuclides with a long half-life from the reactor in Vinča, Serbia, which was built in 1959. Later, short-lived radionuclides were prepared in radiopharmaceutical laboratories in imported generators.^1^

Today, nuclear medicine offers diagnostic services to numerous medical specialties: endocrinology, cardiology and angiology, haematology, nephrology, gastroenterology, neurology, pulmology, infectious diseases, psychiatry and orthopaedics. Nuclear medicine units are modernly equipped and staff are trained for morphological, functional and laboratory diagnostics in clinical medicine and for the integral diagnostics and therapy of different diseases. They work on the treatment of cancer, increased thyroid function and other diseases. Because of the interdisciplinary incorporation of nuclear medicine in diagnostics and treatment, this specialty has an important role in medicine.

## Nuclear medicine in the world

The accidental discovery of radioactivity (Henri Becquerel, 1896) and the research work of the Curies who discovered new radioactive elements, enriched medicine with new therapeutic possibilities based on the effects of radiation. They found that radiation permanently damages live cells. Thus, at the beginning of the 20^th^ century, Alexander G. Bell suggested tumours could be treated with radiation sources. In 1905, radiation was already being used to treat the thyroid gland; however, the broader application of radioactive substances was delayed until the 1920s and 1930s because of their difficult availability. The first experiments were performed with radioactive phosphorus, which accumulates in bones (^32^P) and with iodine (^131^I).

In 1938, a radionuclide, which represents the basis of nuclear medicine, namely technetium – ^99m^Tc was discovered. It has ideal characteristics for use in examinations in humans: a short half-life (6 hours), low radiation dose and the element is not chemically active. It is still used today in 90% of nuclear medicine examinations. In 1939, strontium (^89^Sr) was used for the first time: it accumulates in bones and is still used for pain therapy in patients with bone cancer or bone metastases. A series of new radioactive substances were acquired after the discovery of cyclotron in the beginning of 1940. In 1941, patients were already treated with radioactive iodine (^131^I).

Real progress in the medical application of radioactive substances was achieved by researchers in the fifties with the development of the technetium generator and gamma cameras (Hala O. Anger, 1956). Gamma cameras follow the motion of radioactive substances in the body so that blood flow can be seen and the observation of kidney, thyroid gland or liver functioning becomes possible. Gamma cameras were the first step in the development of tomographic techniques for scanning the human body. New radioactive markers for studying the liver, brain and lungs and radioactive-labelled antibodies were introduced. In the 1970s, measurements of thyroid hormones, insulin, cortisol and pituitary hormones by radioimmune methods were performed.

In hospitals, departments for radionuclide diagnostics were set up, and clinical activity was introduced. Nuclear medicine imaging techniques gradually enabled the localization of tumours, inactive tissues in organs and the estimation of the functional capacity of organs. Diagnostics were constantly changing by combining these investigations with classic methods, and more efficient computers also made a difference. In the 1980s, monoclonal antibodies were introduced. They are useful for discovering cancer cells and for the follow-up of cancer. In the beginning of the 21^st^ century, nuclear medicine is opening new chapters in the diagnostics and treatment of different diseases. Thanks to nuclear medicine, a virtual presentation of organic systems at the molecular level, better knowledge about body functions and a better understanding of diseases and treatment have become possible.

## Nuclear medicine in Slovenia

The beginnings of nuclear medicine in Slovenia date back to 1952, when the Jožef Stefan Institute (JSI) and the Society for Natural Sciences prepared the first lectures for physicians about the application of radioactive isotopes in medicine and later the JSI offered practical courses on working with isotopes. In 1954, Dr Jože Satler (1919–1993) was the first specialist in Slovenia to use radioactive ^131^I for thyroid gland investigations at the Internal Clinic in Ljubljana. He measured the uptake of ^131^I in the thyroid and ^131^I in the urine of three patients with thyroid disease and one healthy volunteer. In the fifties, orthopaedist professor Bogdan Brecelj (1906–1986) treated ankylosing spondylitis at the Orthopaedic Clinic in Ljubljana with the isotope of thorium (^234^Th).^2^ Between 1954 and 1960, the pioneers of nuclear medicine in Slovenia intermittently used ^131^I and ^32^P. Until 1960, when the first laboratory for the needs of nuclear medicine in Ljubljana was built in the backyard of Šentpeter barracks at the Medical Faculty, they didn′t have their own working space.

In 1955, the nuclear medicine team was very small and consisted of Dr Bojan Varl (1920–2002), who continued the work of Dr Satler, and the medical technician Mirko Rozman. Dr Varl systematically introduced the activity of nuclear medicine in improvised measuring rooms of the JSI. Later, Dr Varl and the technician adapted different rooms at the Internal Clinic and changed them into measuring rooms. For nuclear medicine measurements, the JSI loaned a Geiger-Müller detector for *in vivo* measurements with a binary and a decade impulse measuring device. In 1954, this detector was replaced by a detector with a scintillation crystal; the Geiger-Müller tube for the measurement of liquid radioactivity was in use until 1960. They performed the test of ^131^I uptake in the thyroid gland, the two-phase radioiodine test and manual scanning of the thyroid gland. For the first time, they measured the volume of blood and erythrocytes with ^32^P. ^32^P was also used for the treatment of polycythaemia rubra vera and chronic lymphocytic leukaemia. The first radioiodine resections of hyperthyroid goitres were also performed using ^131^I. The first reports on the results of nuclear medicine diagnostics and treatment were published in 1956, 1958 and 1960.

In 1955, nuclear medicine activity also began at the Institute of Oncology in Ljubljana. Dr Leo Šavnik (1897–1968) treated some patients with ovary carcinoma with intraperitoneal injections of colloid gold (^198^Au-colloid). In 1957, Dr Stojan Plesničar began diagnosing and treating thyroid cancer with radioactive iodine. When introducing radionuclides into research, diagnostics and treatment they had to combine and rationalize their efforts. Therefore, in 1957, the Republic’s Centre for the use of Radioactive Isotopes in Medicine was established. The Centre was composed of the Institute of Medical Sciences at the Slovenian Academy of Sciences and Arts, the Internal Clinic, the Orthopaedic Clinic, the Institute of Oncology, the Institute of Pathophysiology and the Institute of Physics at the Medical Faculty of Ljubljana.^3^

In 1959, the Base Laboratory for work with isotopes was built in Ljubljana. It was situated in the extension of the Šentpeter barracks, where the Institute of Oncology also had its rooms. It was opened in February 1960. But it was only really the radiological and isotopic department of the Institute of Oncology, managed by Professor Marjan Erjavec, and the radioisotopic laboratory of the Internal Clinic that actually operated. In the 1950s, several experiments with thorium-X were performed and later abandoned at the Clinic for Orthopaedics. In 1957, at the Institute of Pathophysiology at the Medical Faculty, experiments in which sodium (^22^Na) and potassium (^42^K) ions travelled across cell membranes were begun. These studies were later carried out in their own laboratory.

At the Radioisotopic Laboratory of the Internal Clinic, the permanent medical staff consisted of two internal medicine residents, three technicians and a nurse. The Laboratory gradually adopted essential nuclear medicine equipment: scintillation detectors with electronic counters and printers for *in vivo* clinical studies, a manual scintillation detector with a borehole, a multi-tube Geiger-Müller counter for measurement of radioactivity of urine and faeces in resorption studies, a scanner for electrophoretic and chromatographic bands and, finally, an automatic system for the scintigraphy of organs and for the automatic measurement of gamma radioactivity of liquids. Work has progressed especially since 1962 when the device *Nuclear Chicago* was acquired. The work of the laboratory was expanded and since 1967 when the Renalthron was purchased, the number of renal examinations increased remarkably.^4^

The main activity of the Radioisotopic Laboratory was clinical and outpatient thyroidology. They performed the two-phase radioiodine test, manual and automatic scanning of the thyroid gland, the TRH test, the suppression test and the treatment of hyperthyroidism with radioiodine. In that period they also introduced to standard laboratory work haematological examinations with chromium – ^51^Cr and iron – ^59^Fe, radionephrography with hippuran ^131^I, functional examinations of liver with colloid gold – ^198^Au and Rose Bengal ^131^I, scintigraphy of the liver with ^198^Au, scintigraphy of the pancreas with selenium – ^75^Se-methionin and scintigraphy of the spleen with erythrocytes – ^51^Cr. Blood volume and the lifetime of erythrocytes were measured with autologous ^51^Cr erythrocytes, and at the same time, the splenohepatic index was determined. In that period, the first measurements of fat absorption (^131^ I-olive oil) and vitamin B_12_ were performed and the determinants of iron metabolism were established.

From the start, the annual number of examinations and therapeutic applications rose rapidly. In 1958, all the members of Ljubljana’s centre applied radioactive isotopes for diagnostic or treatment purposes to only 140 to 150 patients. In 1964, the Radioisotopic Laboratory alone performed 1,750 examinations.^4^ In 1968, the laboratory was renamed the Radioisotopic Department of the Internal Clinic. The rapid development of nuclear medicine after 1968 brought a new quality of work and new examinations. This period was marked by the introduction of new radiopharmaceuticals labelled with ^99m^Tc, the scintillation gamma camera and computer management of biomedical data from the gamma camera, inclusion of medical technologists and specialists in nuclear medical work. Organothropic indicators labelled with ^99m^Tc replaced medium-lived indicators, which are more toxic and have a higher energy. New indicators enabled the development of functional investigations with the possibility of computer processing: in gastroenterology the work was taken over by Janez Šuštaršič, in nephrology by Boris Kastelic, in haematology by Nataša Budihna, in pulmonology by Jurij Šorli, in neurology by Franc Hrastnik and in cardioangiology by Miran Porenta. In haemodynamic investigations xenon ^133^Xe became an important radionuclide.^3^

The gamma camera with a processor or with a mini-computer system enabled the development of demanding investigations, such as the measurement of the regional functioning of the lungs, heart, liver and kidneys. They used organ scanning methods in a certain selective physiological phase, methods of filtering unwanted structures in radioisotopic pictures and subtraction methods. With the multi-tube device it was possible to measure the minute heart volume, hippuran kinetics and the velocity of changes in other substances in different organs. In 1970, 7,500 examinations were performed in two chemical laboratories, three measuring rooms, one outpatient department and one haematological laboratory at the Radioisotopic Department.

In 1972, the method of sequential brain angioscintigraphy was introduced. The method enabled the estimation of the vascularization of pathological processes in the brain. In the case of the occlusion of large neck or intracranial arteries, they could detect the decreased level of radioactivity in the affected vascular region. This made possible the distinction between vascular and expansive brain processes.^5^

In 1968, the internists and in 1970 the Radiological Department moved from the Institute of Oncology and only the Isotopic Unit of the Institute of Oncology, known as Izotopi, remained in the rooms of the Base Laboratory. In the 1970s, when other laboratories in Slovenia performed the above examinations, the Isotopic Unit continued its pioneering work. The specialities of this unit, under the guidance of Professor Marjan Erjavec, were: the introduction of new radiopharmaceuticals for bone scintigraphy, the development of new methods such as skin-vein graft perfusion scintigraphy, and the development of computer programs for the analysis of scintigrams.^4^

In 1968, the pioneering work was finished and the development of nuclear medicine departments in general hospitals outside Ljubljana began – first in Slovenj Gradec (1961) and Celje (1968). In the 1970s, the hospitals in Maribor, Izola and Šempeter pri Gorici also acquired their own radioisotopic units.^4^

Progress was made in Ljubljana too. In 1971, the Radioisotopic department moved to the fifth floor of the new building at the Medical centre (MC). It became organizationally independent and in 1972 was renamed the Institute of Nuclear Medicine. In 1973, the Institute moved to its own rooms on the ground floor of the new MC and was again renamed the Clinic of Nuclear Medicine, the present-day Department of Nuclear Medicine.

## Nuclear medicine in Slovenian hospitals

Experts from the Clinic of nuclear medicine had a professional impact on the development of nuclear medicine in all of Slovenia. At the nuclear medicine unit in Slovenj Gradec General Hospital, the pioneer of nuclear medicine was Professor Ivo Raišp (1926–2009), who started performing thyroid gland examinations with radioactive iodine.^6^ He measured the uptake in the thyroid gland and the radioactivity of urine in different time intervals following ingestion of the test dose of ^131^I-sodium iodide. With the new machine acquired in 1968, diagnostics were started. In 1984, a team of 6 people performed 1,446 *in vivo* examinations, 7,212 *in vitro* examinations and treated 10 patients with radioactive iodine. At the nuclear department of the Centre for treatment of internal, infectious and skin diseases at Celje General Hospital, regular work in the newly built Laboratory for nuclear medicine began in 1968 under the direction of primarius Franc Fazarinc. In 1975 they bought a three-tunes device, used mainly as a renograph. Later this device became a constituent element of the equipment of numerous laboratories for nuclear medicine in the former Yugoslavia. The next developmental step was connected with the purchase of a gamma camera DYNA 11/4 with a computer. In 1984, a team of 15 workers performed 5,995 *in vivo* examinations, 43,669 *in vitro* examinations and 17 patients were treated with therapeutic doses of radionuclides.^4^ In Maribor General Hospital, a radioisotopic laboratory was opened in 1973 at the department of internal medicine. The new profession was introduced by Dr Rudi Turk and his five co-workers.^7^ In 1984, 14 co-workers performed 9,731 *in vivo* examinations, 16,978 *in vitro* examinations and treated 36 patients with therapeutic doses of radiopharmaceuticals.^4^ The nuclear medicine unit in Izola began work in 1974. In a small space they had a common working room, an outpatient department, a “hot laboratory”, two measuring rooms for *in vivo* investigations and a room for the application of radiopharmaceuticals and radiochemistry. Alongside Dr Andrej Malej, the unit also employed five technical co-workers on the day of opening. The unit was visited on a weekly basis by professor Erjavec from Ljubljana and primarius Fazarinc from Celje. In 1984, the team comprised eight people who performed 3,110 *in vivo* examinations, 6,159 *in vitro* examinations and eight patients were treated with therapeutic doses of radioiodine. In the General Hospital in Šempeter pri Gorici, the founder of the department for endocrine diseases and nuclear medicine was Dr Bogdan Gornjak, who in 1974 (in Ljubljana) completed the first postgraduate course in nuclear medicine. The unit had a four-channel device, soon they acquired a semiautomatic measuring tool, the *AMES-Gamacord II* and in 1975, the *Nuclear Chicago*. Besides renographies, they also performed scintigraphy of the thyroid gland, kidneys and liver. In 1984, six co-workers of the department performed 2,042 *in vivo* examinations, 3,521 *in vitro* examinations and 11 patients were treated with therapeutic doses of radioiodine.^4^

## Nuclear medicine at the University Medical Centre Ljubljana

In 1973, the Clinic of Nuclear Medicine had nine internal medicine specialists, nine biotechnicians and 16 technical assistants. There were three radiochemistry laboratories with an annual capacity of 20,000 competitive radiochemical investigations and five measuring rooms for *in vivo* measurements, in which 10,000 patients were examined yearly. They performed static and dynamic scintigraphies with quantitative computer-assisted data analysis.^4^

For *in vivo* investigations, in 1973, they had two gamma cameras directly connected to the computer, a device for kidney and liver examinations and an automatic system for the measurement of liquid radioactivity. During the same year, they started measurements of the regional brain blood flow; this was possible because they had computer equipment for processing scintigraphic data. The method was based on the quantitative measurement of clearance of ^133^Xe, injected into the internal carotid artery, from the brain. The investigation was connected with the carotid angiography. In the cysternography, irregularities of the cerebrospinal liquid flow were measured. The examination was performed with the application of technetium, bound to human serum albumin, injected into the lumbar spine channel. On the scintigrams, the distribution of radioisotope in the liquor space in the brain was observed at appropriate time intervals.^5^

Nuclear medicine also spread to pulmonology: perfusion lung scintigraphy, performed with a scanner after the application of a macrocolloid of iron hydroxide, labelled with an Indium isotope ^113m^In, was replaced by perfusion photoscintigraphy on the gamma camera following the application of colloid particles of human serum albumin, labelled with ^99m^Tc. They also introduced the study of lung ventilation with ^133m^Xe and the computer-performed examination of regional ventilation and perfusion.

Nuclear medicine was also introduced into cardioangiography. A few methods with a low clinical value were developed due to insufficient equipment. Computer systems acquired later enabled the representation of heart cavities and large vessels, the calculation of the passage of an indicator through the heart cavities and a presentation and validation of left ventricle contractility. They also adopted radioisotopic coronarography and myocardium scintigraphy. Radioisotopic venography became a standard method for the detection of phlebothrombosis, while simultaneous lung scintigraphy enabled detection of pulmonary embolism.^3^

Investigation of hepatobiliary secretion with ^131^I Rose Bengal rendered possible the distinction between intra- and extrahepatic cholestasis. The introduction of the radiopharmaceutical ^99m^Tc Solco HIDA and computer analysis of the measured data from the gamma camera made it possible to determine indicators of tracer transport in several liver regions and in the intrahepatic and extrahepatic bile ducts. Contrast sharpness on the photoscintigrams of the liver (and lungs) has improved with computer scintigraphy in the inspiration and expiration phases.

Looking further into the past, in 1968, routine pancreas diagnostics with ^75^Se-methionin were already being performed. From the very beginning, the dual radioisotopic technique (^75^Se-methionin and ^198^Au-colloid) and the graphic subtraction of scintigrams was used. Using these radiopharmaceuticals for liver scintigraphy and with the possibility of computer analysis of scintigrams, the method was improved, enabling morphological and functional evaluation of the pancreas.

Functional tests of the urinary system have been performed since 1962. First, radioisotopic nephrography with ^131^I hippuran for differential evaluation of individual kidney function was introduced. In 1965, it was joined by kidney scanning for localisation diagnostics. From 1962 to 1965, the effective plasma flow in kidneys was semiquantitatively evaluated during nephrographic investigations, with measurements of decreasing radioactivity over the precordium (retention index of ^131^I hippurane). In 1965, the evaluation became quantitative (total clearance of ^131^I hippurane), when the quantitative measurement of glomerular filtration (^51^Cr-EDTA clearance) was introduced. Both clearances were performed after a single intravenous injection of the test substance and the catheterization of the urinary bladder was unnecessary. Amongst the first investigations on the gamma camera in 1972, sequential kidney scintigraphy was introduced for the combined diagnostics of focal and segmental kidney diseases, for the evaluation of vesicoureteral reflux and for the radioisotopic angiography of kidneys. Between 1976 and 1977, the computer system for the analysis of measured data from the gamma camera was improved, enabling an automatic and reliable calculation of the total clearance and of the single clearance of ^131^I-hippurane for each kidney. The results of separate renal clearances and plasma renin activity in the renal vein of a single kidney, both introduced in 1975, became important indicators of renal function and decisive factors in the indication of surgical treatment for renovascular hypertension.^3^

Already at the Clinic’s previous location, different substances (mainly hormones) were being measured with analytical methods using radioactive isotopes and by labelling substances with radioactive isotopes for the preparation of indicators, used for *in vivo* investigations. In the first years of the Clinic’s existence, they were mainly determining thyroid hormones using paper chromatography, ionic-exchanging bitumen and dialysis for the separation method and ^131^I as a radioactive isotope. Competitive radioimmunomethods enabled the determination of the following substances: thyroxine (1969), insulin (1969), cortisol (1971), growth hormone (1971), gonadothropins (1972), angiotensin (1973), vitamin B12 (1973), Australia antigen (1974), aldosterone (1974), testosterone (1975), ACTH (1975), TSH (1975), triiodothyronine (1975), prolactin (1975), C-peptide (1976), digoxin (1977) and progesterone (1977).^3^

The radioisotopic determination of plasma hormones enabled clinical endocrinology to make tremendous progress. The determination of plasma hormones in basal conditions and after the stimulation or suppression of the endocrine glands or the hypothalamic-pituitary system made possible the detection and determination of endocrine gland diseases in the latent and manifest phases.

Thyroid scintigraphy and scintigraphy of the adrenal glands in basal, stimulative and suppressive conditions enabled the morphological diagnosis of the process. Nuclear medicine technology mastered endocrinology because it was able to provide objective data about the functioning and morphology of glands.

In 1972, a two-semester postgraduate study of nuclear medicine was introduced at the Department of Nuclear Medicine. In 1974, the course was completed by 10 students.^8^ This programme is being carried out to this day.

In 1975, the clinic had 37 employees. In the same year, the number of *in vivo* investigations was reduced from 10,000 to 8,100 and the number of *in vitro* investigations from 20,000 to 10,000 per year due to lack of space. In the basement of the Outpatient Department, the Department for Thyroid Diseases was set up and in 1978 it evolved into a separate department for outpatient activities with 14 rooms. The Clinic for Nuclear Medicine had three units: the Department for Nuclear-medicine Chemistry, the Department for Nuclear Medicine and the Department for Thyroid Diseases and Nuclear-medicine Endocrinology.

In the 1980s, the Department of Nuclear-medicine Chemistry, managed by chemist Dr Silvester Kladnik (born 1942), annually performed 144,000 investigations. The Department of Nuclear Medicine, which performed the whole diagnostic programme in the field of functional imaging diagnostics of the brain, bones, lungs, heart, liver and kidneys, annually performed 5,400 diagnostic investigations under the guidance of Dr Jurij Fettich (born in 1951) in the same period. The Department for Thyroid Diseases and Nuclear-medicine Endocrinology, which performed outpatient dispensary work for the entire Ljubljana region, performed 20,000 patient examinations in the year 1987 under the guidance of Professor Sergej Hojker (born in 1949).^9^ In that period, the head of the clinic was Professor Miran Porenta (born in 1936).

In 1988, the hospital department of the University Clinic of Nuclear Medicine was opened on the third floor of the Medical Centre. It had six beds for patients with thyroid diseases. The average percentage of occupied beds was 68.3%. The Clinic employed 54 professionals and others, including six Masters of Science, three Doctors of Science, two Medical Faculty employees and one chemical technician on probation.

In the 1980s, the Department of Nuclear Medicine reached its present state, in which progress in the profession is no longer displayed through growth in the number of diagnosed and treated patients, but in the introduction of new diagnostic and therapeutic methods, which are not accessible to other medical branches.

In the beginning of the 1990s, the clinic followed European trends, abandoned out-of-date investigations and introduced new ones. From 1987 to 1991, the number of planned brain scintigrams decreased from 523 to 83 due to the new nuclear-medicine investigation for regional blood-flow, ultrasound and computer tomography. In the same period, the use of radioactive xenon increased. From 1987 to 1989, the number of brain blood-flow investigations increased from 195 to 358. Methods from the early days of nuclear-medicine cardiology such as the estimation of transit times and radioisotopic coronarography were abandoned. Once popular, radionuclide ventriculography decreased markedly due to ultrasound methods. On the contrary, the number of investigations for the estimation of heart muscle perfusion – thallium myocardium scintigraphy – rapidly increased. In 1987, 268 examinations were performed, whereas in 1991, there were 504 examinations. According to the population morbidity, these numbers could have been higher, but there were limitations in purchasing radioactive substances. Thyroid scintigraphy with ^131^I was almost abandoned due to the disadvantageous physical characteristics of this isotope and replaced by scintigraphy with ^99m^Tc and ultrasonography. The number of nuclear-medicine investigations in children has increased. Of all investigations in Slovenia, 67% were performed at the Department of Nuclear Medicine.^10^

In October 1998, the renovated tract of the Department for Nuclear Medicine Diagnostics was opened. In 2011, the clinic under the guidance of Professor Sergej Hojker consists of the following wards: the Department for Nuclear Medicine Diagnostics, the Department for Thyroid Diseases, the Department for Radiopharmacy and the Department for Clinical Radiochemistry.

In 2003, 8,047 scintigraphic investigations were performed in the clinic, 1,468 of them bone scintigraphies. Approximately one third of the investigations were performed on patients who were in the diagnostic procedure because of cancerous diseases. Out of 6,667 patients who were examined for the first time because of thyroid disorders, the fine needle aspiration biopsy and cytologic analysis was needed in 1,326 patients, among which 49 had cancerous changes.^11^

In 2006, 9,276 scintigraphies were performed at the Department of Nuclear Medicine. These included 1,616 bone scintigraphies, 1,054 lung scintigraphies, 1,152 different myocardium scintigraphies, 868 different kidney scintigraphies. The activities of the Department for Thyroid Diseases included 6,973 first examinations, 6,638 control examinations, 2,654 triages, 2,936 consultations, 3,122 thyroid scintigraphies, 7,854 thyroid ultrasound investigations, 1,399 ultrasound-guided thyroid biopsies and approximately the same number of cytological analyses. Around 700 patients were treated with radioiodine and 13 patients were treated with yttrium injection into the joints. The Department for Radiopharmacy and the Department for Clinical Radiochemistry prepared radiopharmaceuticals for the execution of different scintigraphic investigations and for treatment with radiopharmaceuticals. They performed 226,958 laboratory measurements of which the following were the most significant: TSH and thyroid hormones - 81,435, antibodies TPO – 8,960 and antibodies Tg - 9,252, thyroglobulin – 5,930, neo TSH – 19,765 and PKU neo – 20,780, measurements of parathormone - 7,017, cyclosporine – 8,425 and cortisol – 4,992. At the clinic, intensive scientific-research work is performed. In the period between 1996 and 2006 alone, around 250 articles were published in significant journals and have often been cited. The clinic leads national research projects, cooperates in international research projects and introduces new methods of work into the profession. Additionally, it takes part in university teaching, holding lectures, seminars and training in graduate and postgraduate studies. Almost every year, a postgraduate course in nuclear medicine for physicians, pharmacists, chemists, medical physicists and graduates from the high school for health and engineers of radiology is held. Staff also lecture elsewhere. They published a Slovene textbook for health schools *Internal diseases*,^12^ which has been reprinted six times. They have also published contributions in the Slovene textbooks *Endocrinology*,^13^ *Internal medicine*^14^ and *Transplant activity – a donor programme*.^15^ They also participate as members of editorial boards of the national journal *Radiology and Oncology* and of various international journals: *Nuclear Medicine Communications, International Journal of Nuclear Medicine, World Journal of Nuclear Medicine and Hellenic Journal of Nuclear Medicine.*

In recent years, besides the SPECT (single photon emission computed tomography) method, which is combined with CT, positron emission tomography (PET) has also been available which may also be combined with CT (PET-CT).^16^ In Slovenia, we have currently two PET-CT machines (at the Institute of Oncology and at the Department of Nuclear Medicine at the University Medical Centre in Ljubljana). By using short-lived isotopes, accumulated in the metabolically active tissue, they enable the early diagnosis of malignant and other diseases and also enrich cardiovascular diagnostics. Combined with CT, it allows for the acquisition not only of functional but also of excellent anatomical information.

With the development of new radiopharmaceuticals, which bind to specific receptors and to specific antigens, the determination of tissue nature or pathological processes is possible. In this way nuclear medicine has become more precise. In recent years, the use of radiopharmaceuticals for the treatment of malignant diseases, especially lymphomas, neuroendocrine tumours, bone metastases and also for the treatment of rheumatological diseases (such as radiosynovectomies) is increasing. One of the latest discoveries in the field of treatment with radiopharmaceuticals is the use of alpha emitters, which achieve the effect inside the target cell.

## Conclusions

Half a century of development of nuclear medicine has firmly established its status as a branch of medicine with its own processes of diagnostics and treatment. Its past development has been rapid and it is likely that the future will bring new discoveries leading to new methods of work, new equipment, and therefore more effective and safer diagnostic and therapeutic approaches. Likewise, through further education and the acquisition of new knowledge, medical staff will provide the highest possible support in the treatment of patients.

## References

[1] Šuštaršič J (1992). The history of nuclear medicine in Republic of Slovenia. Radiol Oncol.

[2] Varl B (1994). Nuklearna medicina (Nuclear medicine). Enciklopedija Slovenije.

[3] Varl B, Jerše M, Kos M (1978). Nuklearna medicina od ustanovitve na bivši Interni kliniki do danes (Nuclear medicine from its founding on Internal Clinic to nowadays). Tav arjevi dnevi.

[4] Šuštaršič J (1994). Zgodovina nuklearne medicine v Sloveniji (The history of nuclear medicine in Republic of Slovenia). Radiol Oncol.

[5] Varl B (1976). Razvoj Klinike za nuklearno medicino v Ljubljani. (Clinic for nuclear medicine in Ljubljana). Zdrav Vestn.

[6] Plešivčnik D (1996). *Splošna bolnišnica Slovenj Gradec: sto let*. (General hospital Slovenj Gradec – 100 years)..

[7] Puklavec L, Toplak C (2001). Oddelek za nuklearno medicino (Department for nuclear medicine). 200 let SB Maribor.

[8] Šuštaršič J (1974). Poročilo o prvem podiplomskem študiju iz nuklearne medicine v Ljubljani (Report on the first postgraduate study of nuclear medicine in Ljubljana). Zdrav Vestn.

[9] Juras S (1987). Klinična nuklearna medicina naj dobi novo vsebino (Clinical nuclear medicine gets its new content). Bilten KC.

[10] Porenta M, Budihna N, Berginc D, Pavlin K, Milčinski M (1992). Nuklearna medicina v Sloveniji leta 1992 (Nuclear medicine in Slovenia in the year 1992). Med Razgl.

[11] Klinika za nuklearno medicino (Clinics of nuclear medicine) http://www.kclj.si/nuklearna/kdo.htm.

[12] Varl B (1968). *Notranje bolezni: učbenik iz interne medicine za medicinske sestre*. (Internal diseases: textbook for nurses)..

[13] Kocijančič A (1981). *Endokrinologija*. (Endocrinology)..

[14] Kocijančič A, Mrevlje F (1993). *Interna medicina* (Internal medicine).

[15] Avsec Letonja D, Vončina J (2003). Transplantacijska dejavnost: donorski program. *1, Organi*. (Transplant activity – a donor programme).

[16] Hodolic M (2011). Role of (18)F-choline PET/CT in evaluation of patients with prostate carcinoma. Radiol Oncol.

